# Health and the Roof Tree

**Published:** 1923-02

**Authors:** 


THE
### HOSPITAL and HEALTH
REVIEW

### HEALTH AND THE ROOF TREE.

OF the many health problems which press for solution none is more urgent or more vital than that of housing, and every month that passes adds point to its urgency. When Parliament meets towards the middle of February it will be faced by the subject in several forms, all of them acute. Of the housing policy of the new Government the Prime Minister has given us only the barest suggestion, for the sufficient reason that the Cabinet is not yet prepared with its plan. By the beginning of the session, however, it should be ready with proposals covering the whole of a subject which, next to unemployment, affects the health and well-being of the community more closely and more individually than any other. Three Committees are at work upon as many different facets of it. The new Minister of Health, Sir Arthur Griffith-Boscawen, is presiding over a Committee charged with the preparation of a new house-building plan; another is considering the difficulties created by the recent judgment of the House of Lords as to the raising of rents without notice to quit; and a Departmental Committee, which has already presented an interim report, is taking evidence to guide it in suggesting terms upon which the Rent Restriction Act, which will expire in the summer, can be continued without too serious injustice either to house owners or tenants. Each of these aspects of the problem calls for legislation and upon each of them there is likely to be sharp division of opinion, politics having been unhappily allowed to enter into a domain which is primarily economic and sanitary. Leaving party politics aside, we are entitled to say that the Rent Restriction Act as it stands is fair neither to owner nor tenant, and that so long as State interference in this domain continues in any form, the check to house building will continue to a greater or less extent. Free trade in bricks and mortar is essential to a return to conditions enabling the private investor and the speculating builder to resume the activities to which the country owes at least 90 per cent. of its dwellings. The continuance on the Statute-Book of these war-time restrictions creates a feeling of uncertainty and insecurity which arrests the natural flow of capital to the trade of house-building.

Dr. Addison's original housing scheme which ended so disastrously, and has involved the country in a loss of many millions a year for generations to come, was eventually so modified as to restore freedom to the private builder, and the restoration had, within a limited sphere, reasonably satisfactory results. The cost of labour and materials was, however, so great that progress was slow, and the result is that, so far from overtaking the arrears of house building, we have failed even to keep pace with the new demand which accrues from month to month. Unless a carefully prepared plan is put into operation immediately the provision of houses will continue to fall below the demand to an extent so serious that we shall be faced with a crisis that may have lamentable consequence. Human nature is long suffering, but it would be inhuman and impolitic to the last degree to expect a homeless population to endure hardship indefinitely without revolt. All this is clearly recognised by the Government, which has already had statesmanship and common sense enough to realise that the difficulty can only be dealt with by an admixture of official and private action. That is to say, while the fullest freedom will be left to the individual investor and builder, some scheme of State or municipal action, or of the two combined, will have to be adopted. Notwithstanding the lowered cost of materials and the fall in wages, there will be no return to the costly methods which, by strictly limiting local expenditure, placed upon the taxpayer a burden far greater than could be justified. Something has been done to cut the loss thus incurred by allowing local authorities to sell houses they have built, the immediate loss to the Treasury being balanced by an ultimate saving. A certain number of dwellings have thus been disposed of, but the great majority remain in public hands, a heavy and continuous drag upon the finances of the nation.

Although the Government have not yet decided upon their course of action, they are practically bound to proceed upon lines which make the smallest possible demand upon the national finances. With a comprehensive business-like scheme any such demand might be kept at a very low level. When the mixed State and municipal method broke down, the private builder was encouraged to come to the rescue by the grant of a subsidy. This was successful but expensive, and we have now to discover a plan which while attracting capital to the trade of house-building will avoid involving the country in further heavy losses. The most promising plan or series of plans would seem to run upon the lines of making loans to builders and others, either directly from the Exchequer or from the local authorities, or from both. There has been in existence ever since 1889 a Small Dwellings Acquisition Act under which Councils may advance money to tenants for the purchase of the small houses in which they live. The amount of the advance was limited to four-fifths of the value so long as that value did not exceed £400. In 1919 the Act was amended to permit of the local authority advancing 85 per cent. of the cost, and the limit of value was raised to £800. Little advantage has been taken of these powers, and only about a quarter of a million has been advanced, yet it is obvious that the Act might be of the greatest assistance in encouraging new building. If its provisions could be still further extended to embrace the activities of the building societies a new impetus might be given to the provision of housing.

The ideal at which we should aim is that every artisan should be the owner of his own roof tree, but only too often he has been unable to put by money enough to enable him to set the building society in operation. We need a system under which the respectable working man who can produce only a tiny proportion of the cost of building shall be enabled to obtain the balance from a building society or the local authority, the State guaranteeing the lender against the possibility of loss. Under such an arrangement as this the State need not necessarily ever be called upon, and seeing that the purchasers of farms under the Irish Land Acts made practically no defaults we may reasonably take it that the British working man would be equally honest. In any case it would be worth while to run some risk for the sake of the social no less than the economic advantages of such a course. Nothing more certainly contributes to the self-respect of the small man as the proud consciousness that the roof beneath which he dwells is his owb. Tenants who are their own landlords are the best allies of the health officer.

One of the most important departments of housing, the destruction of slums, was necessarily neglected during the war, and during the last four years circumstances have little improved in that respect. We rejoice therefore to know that it is the intention of the Government to widen the area of activity in this direction. Singularly little attention has been paid to the important declaration by Sir J. W. H. Inskip towards the end of last session that it is intended to grant to private enterprise the same powers for the clearing of slums and the rehousing of their inhabitants as have long been possessed by local authorities. Those powers are drastic, and with proper safeguards they might very well be extended to seekers after a profitable commercial adventure. Most municipalities have found that clearances, however necessary for public health and public convenience, are very costly, and if we can get work equally well done by private initiative without cost to the rates, we shall be unwise indeed to neglect the opportunity. The builder and those who finance him have refrained hitherto from turning their attention to slums not because there was no profit in that direction, but because they were debarred by law from enforcing their will upon the owners of the property or upon those who inhabit it. Once individual enterprise in this respect is placed upon the same footing as the local authorities the clearance of slums is likely to proceed with greatly enhanced activity.

Social reformers and professional guardians of public health would view with intense relief so important and far-reaching an amendment of the law. Slums are the worst plague spots of our great towns. They are nursing mothers of disease as well as of vice and crime, and that so many of them should so long have been allowed to continue is a terribly significant comment upon our past disregard of the laws of health and decency and self-respect. Not only do we get rid of slums with painful slowness but, notwithstanding the spread of knowledge of the conditions which make for health, we continue to manufacture them, partly because of the difficulty, which has been growing worse for many years, of providing a sufficiency of sanitary housing. Overcrowding is one of the root causes of the creation of slums; a minor but exceedingly exasperating one is the difficulty of punishing people who cut up staircases for firewood, and mis-use sanitary appliances in such a way as to endanger the health of a whole neighbourhood. Nor must we forget those who deliberately buy leasehold houses, the term of which is approaching, and so exploit them as to derive the utmost possible profit from the necessities of the unfortunate and the vicious alike. Upon speculators of this type we need have no mercy. The rights of property are one thing; the right of an individual to wring the last farthing from rotten tenements, teaming with huddled humanity, no civilised State can recognise. The sooner, therefore we give leave and licence to commercial enterprise to help local authority to replace these breeding-places of tuberculosis and many another scourge, with clean and airy dwellings, the better will it be for the future of a race threatened with the evils which flow from the undue concentration of humanity in overgrown and haphazard towns.

